# Cerebral ischaemia and matrix metalloproteinase-9 modulate the angiogenic function of early and late outgrowth endothelial progenitor cells

**DOI:** 10.1111/jcmm.12116

**Published:** 2013-08-15

**Authors:** Anna Morancho, Mar Hernández-Guillamon, Cristina Boada, Verónica Barceló, Dolors Giralt, Laura Ortega, Joan Montaner, Anna Rosell

**Affiliations:** a Neurovascular Research Laboratory, Vall d’Hebron Research Institute, Universitat Autònoma de BarcelonaBarcelona, Spain

**Keywords:** endothelial progenitor cell, matrix metalloproteinase-9, cerebral ischaemia, angiogenesis, neurorepair, stroke

## Abstract

The enhancement of endogenous angiogenesis after stroke will be critical in neurorepair therapies where endothelial progenitor cells (EPCs) might be key players. Our aim was to determine the influence of cerebral ischaemia and the role of matrix metalloproteinase-9 (MMP-9) on the angiogenic function of EPCs. Permanent focal cerebral ischaemia was induced by middle cerebral artery (MCA) occlusion in MMP-9/knockout (MMP-9/KO) and wild-type (WT) mice. EPCs were obtained for cell counting after ischaemia (6 and 24 hrs) and in control animals. Matrigel™ assays and time-lapse imaging were conducted to monitor angiogenic function of WT and MMP9-deficient EPCs or after treatment with MMP-9 inhibitors. Focal cerebral ischaemia increased the number of early EPCs, while MMP-9 deficiency decreased their number in non-ischaemic mice and delayed their release after ischaemia. Late outgrowth endothelial cells (OECs) from ischaemic mice shaped more vessel structures than controls, while MMP-9 deficiency reduced the angiogenic abilities of OECs to form vascular networks, *in vitro*. Treatment with the MMP inhibitor GM6001 and the specific MMP-9 inhibitor I also decreased the number of vessel structures shaped by both human and mouse WT OECs, while exogenous MMP-9 could not revert the impaired angiogenic function in MMP-9/KO OECs. Finally, time-lapse imaging showed that the extension of vascular networks was influenced by cerebral ischaemia and MMP-9 deficiency early during the vascular network formation followed by a dynamic vessel remodelling. We conclude that focal cerebral ischaemia triggers the angiogenic responses of EPCs, while MMP-9 plays a key role in the formation of vascular networks by EPCs.

## Introduction

Stroke is a major cause of morbidity and mortality worldwide, but the only available treatments are reperfusion therapies using tissue plasminogen activator or clot retrieval interventions in certain patients with acute ischaemic strokes. However, only 2–5% of stroke patients are receiving thrombolytic therapy to restore the blood flow 1 and the impact of clot retrieval interventions is still under evaluation. In this context, as important as neuroprotection therapies, it is the enhancement of endogen neuroreparative responses that contributes to stroke recovery 2. It has been proposed that the activation of angiogenesis and neurogenesis in the perfused peri-infarct areas might remodel the damaged frontier tissue leading to behavioural recovery 3. For that reason, the enhancement of endogenous angiogenesis after stroke, which can be activated with cell-based therapies, is considered to be crucial in future neurorepair strategies.

Angiogenesis is defined as the growth of new blood vessels from pre-existing vascular structures and EPCs have been shown to participate in neovascularization in the adult 4. Circulating EPCs can be mobilized endogenously in response to ischaemia, home to sites of neovascularisation and differentiate into endothelial cells, becoming a new model for endothelial generation and vessel repair 5. No single marker exists for this population of progenitor cells and it is accepted that at least two subsets can be obtained under cell culture: early EPCs, which appear early in cultures with spindle-shaped morphology (also named circulating angiogenic cells) and OECs, which appear later and present clonogenic and tubulogenic potential 6–9. Several studies using *in vitro* and *in vivo* models have demonstrated the role of EPCs as a pro-angiogenic cell-based treatment in hindlimb or cerebral ischaemia 10–13. The factors influencing EPCs function are still being identified and under investigation as their modulation might improve future cell-based therapies.

During new vessel formation, one of the earliest steps is the degradation of the basal membrane, and MMPs are key players that could determine the success of this complex process 14. Among them, the gelatinase MMP-9 has been shown to be essential for capillary branching, invasion and tube formation of endothelial cells 15–16. Additionally, MMP-9 has been shown to play a dual role after ischaemia, their up-regulation being detrimental in the acute phases, but becoming essential for an effective neurorepair 17–21.

Our hypothesis is that cerebral ischaemia is a trigger for EPC release and functions, while MMP-9 deficiency reduces EPC levels and impairs angiogenic function in the context of cerebral ischaemia. For this purpose, EPC cell-culture yields and function were explored in MMP 9-deficient mice compared with WT animals subjected to middle cerebral artery occlusion. We demonstrate that the angiogenic responses of EPCs are enhanced by the ischaemic insult and impaired in the absence of MMP-9. To further test our hypothesis, the function of EPCs from control subjects was also studied in the presence of two MMP inhibitors, demonstrating the key role of MMPs and MMP-9 in the vasculogenic function of EPCs. Time-lapse imaging shows for the first time the patterns of vessel network formation, which are clearly aberrant in MMP 9-deficient EPCs and enhanced in ischaemia-stimulated EPCs.

## Materials and methods

### Animals

Age-matched male mice KO for MMP-9 (MMP-9/KO) and WT mice (strain background FVB) from Jackson Laboratories (Sacramento, CA, USA) were used in this study. Matrix metalloproteinase-9 null mice were generated by replacing part of exon 2 and all intron 2 with a phosphoglycerate kinase-neomycin cassette as described by Vu *et al*. 22. Both mice genotypes (MMP-9^−/−^ and MMP-9^+/+^) were bred in-house and the offspring was used for experimentation. Genotyping analysis was performed as detailed in supplemental data. All animals were housed in a temperature-/humidity-controlled room and maintained on a 12-hr light/12-hr dark cycle. Only adult males (25–35 g bw) were used in the study and free access to food and water was allowed prior and after surgery. A total of 92 mice were initially included for the middle cerebral artery occlusion (MCAO) model (ischaemic and sham animals). All procedures were approved by the local Animal Care Committee and were conducted in compliance with the Spanish legislation and in accordance with the Directives of the European Union.

### Permanent focal cerebral ischaemia

A reproducible model of stroke by occluding the middle cerebral artery (MCA) affecting the cortex was induced as previously described (see Supporting Information), 23 Sham animals underwent all surgical procedures with the exception of the MCA occlusion. Six or 24 hrs after the ischaemia, or after 24 hrs of the surgery in sham group, mice were killed.

### Mouse endothelial progenitor cell cultures

Mouse early EPCs were obtained as previously described 24. Spleens from WT and MMP-9/KO mice were obtained at 6 or 24 hrs after ischaemia and in sham animals after 24 hrs. A total of 38 cell cultures were performed from 76 mice (two spleens each) and images from five representative fields were taken at 200× at day 5. EPCs were defined as spindle-shaped cells at day 5 as previously described and phenotyped 24–25. Cell counts were performed by an investigator blinded to the experimental group. Sub-confluent colonies were cultured for further expansion; those cells, presenting cobblestone or palisade morphology, are named OECs and were used for *in vitro* vessel formation Matrigel™ assays, see Figure S1. Detailed methods are available in Supporting Information.

### Human blood EPCs cultures

Human OECs were obtained as previously described from peripheral blood from healthy controls (aged from 39 to 59) 25; detailed methods are available in Supporting Information.

### Immunocytochemistry

Standard EPC phenotyping was performed in mouse and human OECs for von Willebrand factor, KDR and CD133 antigens. Methods are available in Supporting Information.

### *In vitro* vessel formation

To assess the role of ischaemia and MMP-9 on angio-vasculogenic abilities of OECs, Matrigel™ matrix (BD Biosciences, San Jose, CA, USA) was used for *in vitro* vessel formation (also named tubulogenesis). Experimental groups consisted in mouse OECs obtained from ischaemic (24 hrs) or sham mice, from now on named ischaemic or control OECs, respectively, or human OECs. Additionally, mouse WT and human cells were treated with the MMP inhibitor GM6001 (CC100, EMD Millipore, Darmstadt, Germany) at 10 or 20 μM or the specific MMP-9 inhibitor I (444278, EMD Millipore) at 100 nM for mouse or 0.5 and 1 μM for human cells. Finally, MMP-9/KO cells were treated with conditioned media (CM) obtained from WT OECs or with 20 or 40 nM recombinant mouse pro-MMP-9 (R&D systems, MN, USA) at 20 or 40 nM. Detailed methods are available in Supporting Information. The number of complete rings and the total tube length (perimeter of the complete rings) were counted by ImageJ software (NIH, Bethesda, MD, USA) by an investigator blinded to the treatment. Mean values were used for comparisons between cell types while experimental treatments with MMP inhibitors, CM or recombinant MMP-9 were expressed as percentage of the non-treated group.

### Cell viability

Cell viability assay was additionally performed as previously described 26 to assess the potential toxicity of the MMP inhibitors and their vehicle media applied to the OECs. Detailed methods are available in supporting methods.

### Live time-lapse imaging for *in vitro* vessel formation

The formation of vessel-like structures by mouse OECs was assessed during 24 hrs using time-lapse imaging. Experimental groups were: WT control, WT ischaemic, MMP-9/KO control and MMP-9/KO ischaemic OECs. Standard Matrigel™ assay was conducted as described above and image acquisition started 1 hr after seeding and then every 30 min. up to 24 hrs. An Olympus multi-dimensional-TIRFM cell-R microscope (Olympus, Tokyo, Japan) with temperature, CO_2_ and humidity control was used. Two images per well were acquired at 40× and 100×. Complete rings and branching points (points featuring three or more connections) were counted every 2 hrs by an investigator blinded to the treatment.

### Gelatin zymography

Presence of MMP-9 in CM from WT control and ischaemic cultures was confirmed as described in the supplemental data.

### Brain vasculature quantification

A group of eight WT and eight MMP-9/KO mice were subjected to permanent focal cerebral ischaemia (*n* = 4 WT and *n* = 4 MMP-9/KO) or sham surgery (*n* = 4 WT and *n* = 4 MMP-9/KO). To assess endogenous angiogenesis, after 21 days, mice were injected intravenously with 80 μg of Dylight 594-labelled tomato lectin (*Lycopersicon esculentum*; Vector Laboratories, Burlingame, CA, USA) and sacrificed by cardiac perfusion of 4% paraformaldehyde under deep anaesthesia. Brains were collected and the lectin positive cortex area analysed. Imaged vessels presented a diameter smaller than 10 μm typical of microvessel structures. Detailed methods are available in Supporting Methods.

### Statistical analysis

SPSS 15.0 package was used for statistical analyses. Shapiro–Wilk test was used to verify if variables had a Gaussian distribution. Normally distributed variables are expressed as mean ± SD and represented by bars, statistical significance for intergroup differences was assessed by Student’s *t*-test or anova followed by Tukey post-hoc test. A *P* < 0.05 was considered statistically significant at a 95% confidence level. Extreme values were excluded (mean ± 2SD) (*n* = 3); this criterion only affected early EPC counts.

## Results

### Characterization of MMP 9-deficient mice and EPCs

**Figure 1:**
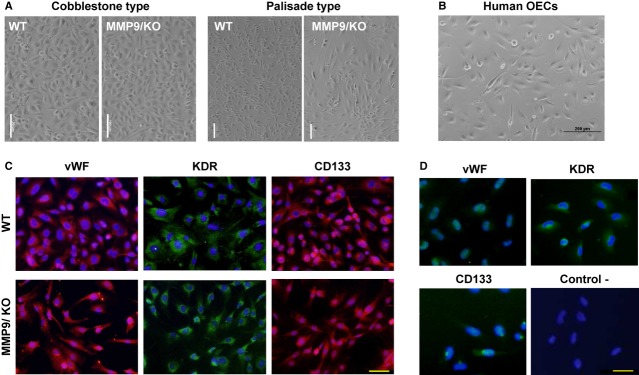
Immunophenotypic characteristics of outgrowth endothelial cells (OECs) from wild-type (WT) and matrix metalloproteinase-9/knockout (MMP-9/KO) mice and human controls. (A) Representative images of OECs from WT and MMP-9/KO obtained from ischaemic mice showing cobblestone or palisade cell morphologies (bar = 100 μm). (B) Representative micrographies from human OECs. Immunofluorescent staining of OECs showing positive signal (red or green) for von Willebrand factor (vWF), KDR and CD133 (bar = 50 μm) in mouse palisade-type (C) and human cobblestone-type cells (D). The blue signal corresponds to nuclear DAPI.

Wild-type and MMP-9/KO genotypes were confirmed by PCR (Fig. S2). A total of 32 mouse EPCs cultures were finally included in the study after discarding extreme values and contaminated cultures. Colonies of expanding OECs were obtained in 6 out of 17 cultures from WT mice (35.3%) and in 2 out of 15 from MMP-9/KO mice (13.3%) displaying two different morphologies: cobblestone- or palisade- type. No differences were seen regardless of mouse MMP-9 genotype in terms of cell morphology (Fig. 1A and B). Immunophenotyping showed that both WT and MMP-9/KO mice and human OECs were positively stained for endothelial markers (vWF and KDR) and CD133 progenitor cell marker (Fig. 1C and D) regardless of morphology type (see additional immunophenotyping in Fig. S3).

### Influence of focal cerebral ischaemia and MMP-9 deficiency on EPC yields

**Figure 2:**
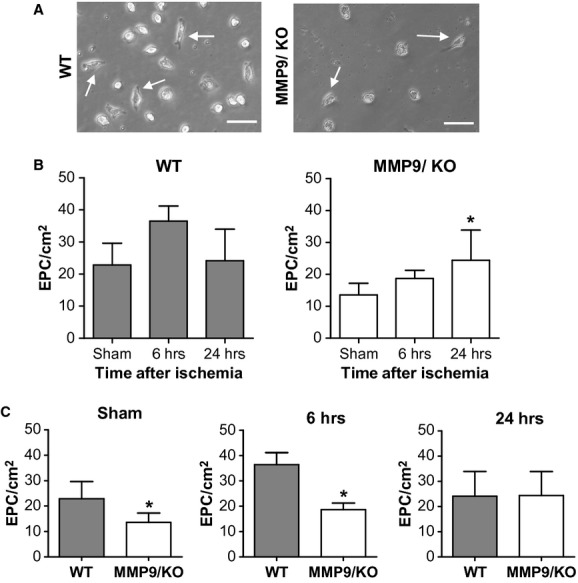
Cell density of early endothelial progenitor cells (EPCs). (A) Representative image of spindle-shaped early EPCs (arrows); bar = 50 μm. (B) Bars representing cell density of EPCs from sham and ischaemic wild-type (WT) or matrix metalloproteinases-9/knockout (MMP-9/KO) mice (anova followed Tukey post-hoc test; *P < 0.05 *versus* sham). (C) Number of EPCs at different time-points of ischaemia in WT and MMP-9/KO mice. (*T*-test; *P < 0.05). Results are expressed as mean ± SD, *n* = 4–6/group.

The number of early EPCs as spindle-shaped cells in cultures was counted (Fig. 2A). Our results showed that in WT mice the number of EPCs was altered by ischaemia (Fig. 2B, *P* = 0.055) and although an increase was detected at 6 hrs this was not significant (*P* = 0.066) nor was the change detected at 24 hrs (*P* = 0.967). Conversely, in MMP-9/KO mice the ischaemic insult altered the complete temporal profile (Fig. 2B, *P* = 0.049) by delaying the peak in the number of EPCs being significantly higher 24 hrs after ischaemia compared with sham animals (*P* = 0.041). Matrix metalloproteinase-9 deficiency strongly decreased the number of EPCs in sham and 6 hrs ischaemic mice compared with WT mice (*P* = 0.012 and *P* = 0.019, respectively); see Figure 2C. At 24 hrs, WT and MMP-9/KO mice presented a similar number of EPC yields (*P* = 0.969) as a consequence of the delayed increase in EPCs after the ischaemic event in MMP-9/KO mice.

### Focal cerebral ischaemia enhances the ability of WT mouse OECs to form vessel-like structures determining the maximum extension of vessel-like networks

**Figure 3:**
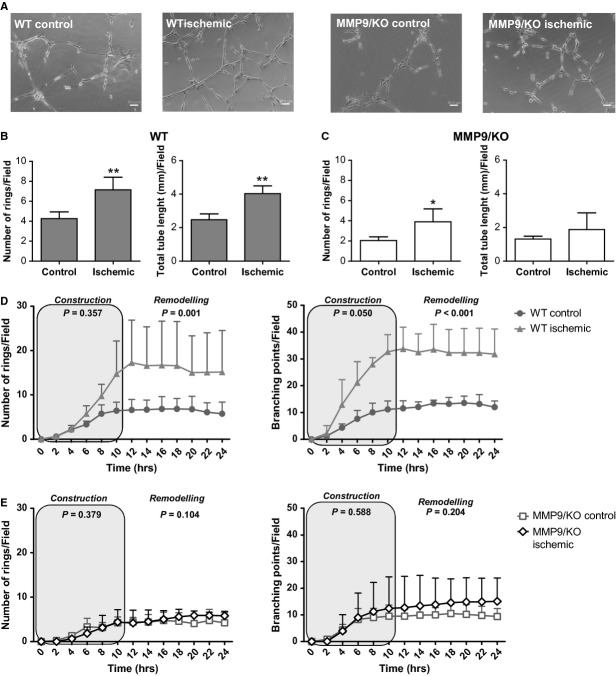
Effect of ischaemia on Matrigel™ assay performance of mouse outgrowth endothelial cells (OECs). (A) Representative micrographs of vessel-like structures shaped by control and ischaemic (24 hrs) wild-type (WT) and matrix metalloproteinases-9/knockout (MMP-9/KO) OECs (bar = 50 μm). The number of rings and total length of vessel-like structures of control and ischaemic OECs was quantified for WT (B) and MMP-9/KO cells (C). Quantification of time-lapse imaging assay of mouse OECs on Matrigel™ matrix shows the effect of ischaemia on WT (D) and MMP-9/KO (E) on the formation and dynamics of vessel-like structures in each experimental group. Two clear patterns were observed in all groups highlighted as construction or remodelling. Results are expressed as mean ± SD; (*T*-test; *P < 0.05, **P < 0.01 *versus* control group, *n* = 3–4 independent experiments).

Figure 3A shows representative images of vessel-like structures shaped by OECs of different studied groups. Quantification of Matrigel™ assays showed that WT ischaemic OECs were able to shape more vascular networks than sham OECs. Ischaemic cells obtained from WT mice completed a higher number of rings (*P* = 0.007) and shaped larger networks (*P* = 0.002) than control cells. In contrast, in MMP-9/KO cells the ischaemic insult only increased the number of rings (*P* = 0.031), but had no effect on the network length (*P* = 0.313); see Figure 3B and C.

Time-lapse imaging revealed that generation of vessel-like structures into a Matrigel™ matrix began only 2 hrs after seeding, while the maximum number of vessel-like structures was shaped approximately at 10 hrs as shown in Figure 3D and E and Figure S4. Interestingly, the construction period was followed by a dynamic remodelling seen only in the supporting video files of time-lapse imaging (Videos S1–S4). Table 1 shows significant differences on vessel network formation during the established construction period *versus* the remodelling period. Time-lapse imaging results confirmed that angiogenic abilities of OECs are strongly enhanced in WT ischaemic cells (*P* < 0.05 *versus* WT control cells). However, in a context of MMP-9 deficiency, ischaemic cells were not superior to control cells showing the importance of MMP-9 for angiogenic responses of EPCs (Fig. 3D and E, Figure S4 and Videos S1–S4).

**Table 1:** Quantification of tube-like structures per hour in the construction (0–10 hrs) and remodelling (10–24 hrs) phases in Matrigel™ assays

Group	Increase in rings/hour			Increase in branching points/hour		
	0–10 hrs	10–24 hrs	*P*-value	0–10 hrs	10–24 hrs	*P*-value
Control WT	0.64 ± 0.5	−0.05 ± 0.5	<0.001	1.12 ± 0.7	0.06 ± 0.7	<0.001
Control MMP-9/KO	0.43 ± 0.5	0.00 ± 0.3	0.005	0.95 ± 1.0	0.00 ± 0.47	0.004
Ischaemic WT	1.48 ± 1.6	0.00 ± 0.9	0.005	3.27 ± 3.1	−0.70 ± 0.8	0.001
Ischaemic MMP-9/KO	0.44 ± 0.4	0.10 ± 0.3	0.005	1.24 ± 1.7	0.19 ± 0.4	0.031

Data are given as increase *per* hour and expressed as mean ± SD.

### MMP-9 deficiency impairs the formation of vessel-like structures by mouse OECs

**Figure 4:**
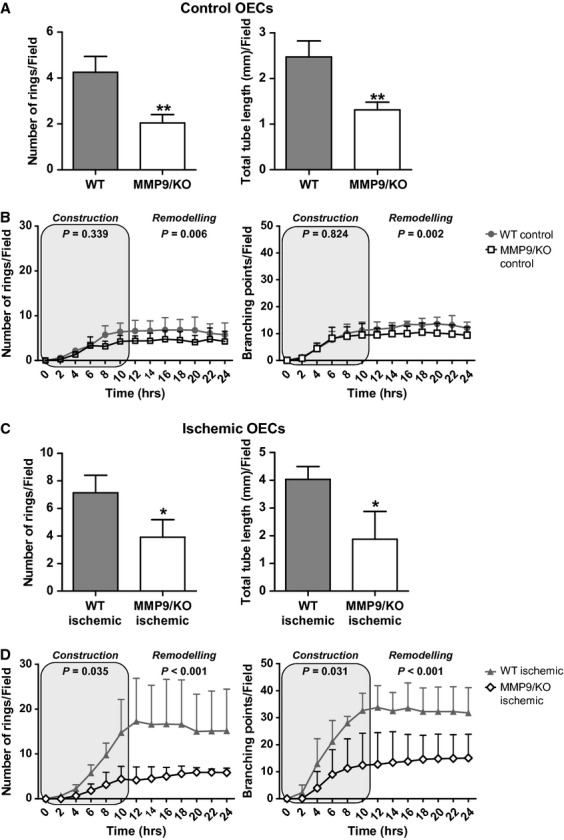
Effect of matrix metalloproteinase-9 (MMP-9) deficiency on Matrigel™ assay performance of mouse outgrowth endothelial cells (OECs). Bar graphs showing *in vitro* tubulogenesis performance of wild-type (WT) control compared with MMP-9/knockout (MMP-9/KO) cells (A) and the corresponding time-lapse imaging (B) and WT and the MMP-9/KO ischaemic OECs (C) and the corresponding time-lapse imaging (D). Results are expressed as mean ± SD. (*T*-test; *P < 0.05, **P < 0.01; *n* = 3–5 independent experiments).

Control cells with MMP-9/KO genotype showed reduced angiogenic abilities when compared with WT cells as demonstrated by less rings and shorter total tube length shaped by MMP-9/KO control OECs in Matrigel™ assays (*P* = 0.001 and *P* = 0.001, respectively); see Figure 4A. Time-lapse imaging confirmed the influence of MMP-9 deficiency in the vessel network formation over time (Fig. 4B). The video images (Videos S1 and S3) also illustrate differences in the patterns of movement, showing that WT OECs can move much faster and make more cell-to-cell connections than MMP-9/KO OECs, which show clear impaired function. The same experimental groups were tested using ischaemic cells as seen in Figure 4C. Ischaemic MMP-9/KO OECs showed impaired angiogenic function compared with WT ischaemic cells by forming less rings (*P* = 0.011) and diminishing total tube length (*P* = 0.016). Figure 4D, Figure S4, Videos S2 and S4 show how the MMP-9 deficiency impairs the vessel network formation over time also in ischaemic cells, demonstrating the importance of MMP-9 for angiogenic responses of EPCs in the context of ischaemia.

**Figure 5:**
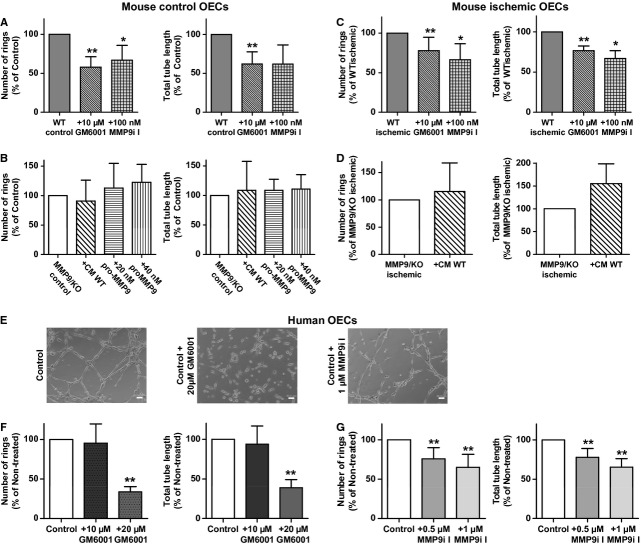
Effect of matrix metalloproteinases (MMP) inhibition on Matrigel™ assay performance of mouse and human outgrowth endothelial cells (OECs). (A) Bar graphs showing impaired angiogenic function of wild-type (WT) control cells when treated with GM6001 and MMP-9 inhibitor I (MMP-9i I). (B) Bar graphs showing that angiogenic performance could not be reverted when MMP-9/knockout (MMP-9/KO) OECs were treated with exogenous MMP-9 contained in conditioned media (CM) of WT OECs or by recombinant pro-MMP-9 treatment. (C) Bar graphs showing the effect of pharmacological MMP inhibition of the angiogenic function of WT ischaemic cells. (D) The addition of CM of ischaemic WT OECS could not significantly revert the angiogenic impairment of MMP-9/KO ischaemic cells. (E) Representative micrographs of vessel-like structures from human OECs in the presence or absence of GM6001 and MMP-9 inhibitor I (MMP-9i I); bar = 50 μm. Bar graphs represent number of rings and the total tube length determined in OECs treated with GM6001 at a final concentration of 10 or 20 μM (F) or treated with 0.5 and 1 μM of MMP-9i-I (G). Results are expressed as mean ± SD and as a percentage of the non-treated group; for different treatments a *T*-test *versus* the non-treated control group was performed in A–D while anova followed by Tukey post-hoc test was performed for dose-response analysis in F and G; *P < 0.05, **P < 0.01; *n* = 5–10 independent experiments.

To further confirm the role of MMP-9, WT control cells treated with the broad spectrum MMP inhibitor GM6001 and the specific MMP-9 inhibitor I significantly reduced their angiogenic abilities by decreasing the number of rings compared to non-treated cells (*P* < 0.001 and *P* = 0.04, respectively) and the total tube length (*P* < 0.001 and *P* = 0.054, respectively); see Figure 5A. The addition of exogenous MMP-9 by adding CM from WT OECs containing MMP-9 (Fig. S5), 20 or 40 nM of recombinant pro-MMP-9 could not revert the ability to form vessel-like structures of MMP-9/KO cells (*P* > 0.05 for ring structures and total tube length for all experimental conditions) as shown in Figure 5B. In addition, the treatment of ischaemic WT OECs with GM6001 and MMP-9 inhibitor I also reduced the angiogenic performance of ischaemic OECs, showing less number of rings (*P* = 0.007 and *P* = 0.044, respectively) and diminished total tube length (*P* = 0.005 and *P* = 0.044) than non-treated cells (Fig. 5C). In MMP-9/KO ischaemic OECs, the addition of CM from WT OECs did not improve significantly the formation of vessel-like structures as shown in Figure 5D (*P* = 0.579 for number of rings and *P* = 0.253 for total tube length).

Any of the MMP inhibitor treatments or their vehicles affected the cell viability of WT control or ischaemic OECs as shown in Figure S6A and B.

### Inhibition of MMPs reduces the angiogenic capacity of human OECs

Human OECs with cobblestone morphology were used in the tubulogenic assays. When treating OECs from control animals with 10 μM of GM6001, we could not observe reduced angiogenesis as seen in Figure 5F. Doubling the concentration of GM6001 to 20 μM led to a significant decrease in OECs angiogenic function by reducing both the number of vessel structures (*P* < 0.001) and total tube length (*P* < 0.001) compared with non-treated OECs; see Figure 5E and F. The specific role of MMP-9 was also demonstrated as treatment with 0.5 and 1 μM of MMP-9 inhibitor I also decreased the number of rings (*P* < 0.001 and *P* < 0.001, respectively) and the total tube length (*P* < 0.001 and *P* < 0.001, respectively).

None of the tested doses of GM6001 or MMP-9 inhibitor I and none of their vehicles showed any effect on cell viability; see Figure S6C.

### *In vivo* angiogenesis after focal cerebral ischaemia is impaired in MMP 9-deficient mice

In a pilot study, we explored how MMP-9 deficiency could affect endogenous angio-vasculogenesis in the context of cerebral ischaemia. When comparing the amount of functionally perfused blood microvessels (as stained by lectin intravenous perfusion) in the peri-infarct cortex between control and ischaemic animals, WT mice showed an increase in the vessel density 21 days after ischaemia in some areas of the cortex affected for the infarct (*P* = 0.026 for posterior areas). However, in MMP-9/KO mice, there was no increase in the vessel density after the ischaemic insult in any of the studied areas (*P* > 0.05); see Figure S7.

## Discussion

Our study shows that focal cerebral ischaemia promotes an acute mobilization of EPCs, while MMP-9 deficiency decreases the number of circulating EPCs delaying their mobilization after an ischaemic insult. We also demonstrate that focal cerebral ischaemia enhances the angiogenic abilities of late outgrowth EPCs *in vitro*, being partially impaired in MMP 9-deficient cells. Finally, the pharmacological inhibition of MMPs, specifically MMP-9, impaired the angiogenic function of OECs, suggesting that MMP-9 is necessary for *de novo* vascular network formation. We also suggest that endogenous angiogenesis in peri-infarct areas after focal cerebral ischaemia is partly inhibited in MMP-9/KO mice. All these data proposes MMP-9 as a key protease in post-stroke angiogenesis mediated by EPCs.

Two different types of EPCs have been described and identified in culture, early EPCs and late OECs, which present differences in phenotype, proliferation capacity and function 7. Recruitment and mobilization of EPCs play an important role in neovascularization after tissue injury. Different studies in stroke patients have shown an increase in circulating EPCs at different time-points after the ischaemic insult, while other authors have described lower levels of EPCs in stroke patients than in controls 25–29. To evaluate these contradictory data, we aimed, for the first time, to assess the mobilization of EPCs after an ischaemic insult in a mouse model of stroke. We have quantified early EPCs in short-term cell cultures from mouse spleens by counting spindle-shaped cells as previously described by other authors 24–25. The results show an increase in the levels of circulating EPCs early after the ischaemic insult. We have also demonstrated that MMP-9 deficiency delayed the ischaemia-induced EPC mobilization. In this sense, it is known that MMP-9 is required for the mobilization of stem and progenitor cells from the bone marrow 30–31. According to these studies, we have observed decreased levels of early EPCs in MMP-9/KO sham mice compared with WT animals, confirming an impaired mobilization in MMP 9-deficient animals. However, as we are considering cultured cells, we have to acknowledge that other factors could be influencing the final counts such as the cell adherence or survival capacities between WT and MMP-9/KO EPCs. This result is in agreement with other findings describing impaired mobilization of bone marrow-derived EPCs in MMP 9-deficient mice 30. In our study, the ischaemic insult restored the impaired EPC mobilization of MMP-9/KO sham animals by increasing EPCs up to the same level as WT mice at 24 hrs. This might suggest that our model of focal cerebral ischaemia activates other triggers for bone-marrow cell mobilization in the absence of this protease or compensatory effects by other MMPs explaining the delayed response to the ischaemic trigger. Our study focuses on the mobilization and function of EPCs during the acute phase of cerebral ischaemia. However, the long-term effects and capacity of these cells to modulate neurorepair in these animal models were not in the scope of the present study and will be investigated in future studies.

In the present study, we could obtain late EPCs from spleen cultures of WT and MMP-9/KO mice, but cultures from MMP 9-deficient animals yielded a lower percentage of outgrowth cells. In this regard, other authors have reported reduced colony forming units of human circulating cells in the presence of an MMP-9 inhibitor 32. As less cell density was found in MMP-9/KO cultures, we can hypothesize that it could influence the reduced yields of outgrowth EPCs as described by other authors 33. Another explanation could be in the nature of MMP-9 deficiency and altered mechanisms of EPCs maturation into an endothelial phenotype. The identification of the molecular mechanisms leading to OECs appearance will provide more information of the MMP-9 role in that process.

Our results demonstrate that the OECs obtained from WT ischaemic mice showed enhanced endothelial function by performing higher number of vessel-like structures than OECs from non-ischaemic mice. On the other hand, this angiogenic function was only partially enhanced in ischaemic MMP-9/KO cells, suggesting a mild effect of the ischaemic trigger on the MMP 9-deficient OECs. The videos supplied as supporting files clearly show the persistent impairment of MMP-9/KO cells to shape vascular networks despite the ischaemic trigger. This strongly indicates that focal cerebral ischaemia can improve angiogenic abilities of OECs and preserve these abilities after maturation into endothelial-like cells, but this trigger is dependent on MMP-9 activity. To our knowledge, this is the first work comparing OECs’ vasculogenic abilities from ischaemic and non-ischaemic animals *in vitro*. Supporting our data, a recent investigation has published that hypoxia pre-conditioned bone marrow mesenchymal stem cells (BMSCs) transplanted in rats after cerebral ischaemia improved the regenerative potential of standard BMSCs, suggesting mechanisms involving enhanced expression and release of trophic/growth factors 34. The factors that cerebral ischaemia specifically activates in circulating EPCs to enhance their angiogenic potential and how the activation is maintained need to be further studied.

We have shown that MMP-9 deficiency and pharmacological inhibition of MMP-9 clearly impair the angio-vasculogenic abilities of OECs and that the addition of exogenous MMP-9 could not reverse it, suggesting that endogenous secreted MMP-9 is essential for a correct EPC function in terms of *de novo* vessel formation. We have further demonstrated that OECs from MMP-9/KO mice built aberrant vascular networks compared with WT cells, suggesting a key role of this MMP in *de novo* vascular formation. Other authors have shown similar results when inhibiting MMP-9 in human control OECs 32. However, we show, for the first time, by time-lapse imaging, the tube formation patterns over time and specific inability of MMP-9/KO EPCS for cell migration and for the establishment of cell-to-cell interactions.

Importantly, we have successfully translated our results into the human setting. Outgrowth endothelial cells from human controls diminished their angiogenic abilities and shaped smaller vascular networks when treated with the broad spectrum MMP inhibitor or with a specific MMP-9 inhibitor.

The function of MMP-9 in vascularization has been previously studied in other diseases. Johnson and colleagues described in a model of hindlimb ischaemia that MMP-9 was necessary for ischaemia-induced angiogenesis as an inhibition of tissue angiogenesis and a decreased capillary perfusion were found in MMP-9/KO mice 15. Conversely, in myocardial infarction, a stimulation of angiogenesis has been described in MMP-9/KO animals, suggesting a possible dual role of this MMP depending on the tissue 35. However, in the brain, MMP-9 seems to be essential for an effective neurorepair 21. It is well described that endogenous activation of angiogenesis occurs within the first hours after cerebral ischaemia 36. Our results support the critical role of MMP-9 in spontaneous angiogenesis after cerebral ischaemia as we have shown an increase in specific peri-infarct vessels in ischaemic WT mice, but not in MMP-9/KO animals when compared with non-ischaemic animals. It is important to highlight that with our perfusion technique of intravenous lectin to stain the blood vessels, we are only considering functional vessels with normal blood perfusion excluding any non-functional aberrant vasculature. Whether the impaired angiogenic function of OECs lacking MMP-9 observed in the present study could influence neurorecovery needs to be elucidated and warrants further studies using *in vivo* models of ischaemia receiving cellular therapy.

Finally, time-lapse imaging technique performed to examine the dynamics of vessel formation in Matrigel™ matrix confirmed that both cerebral ischaemia and MMP-9 determined the extension of the vascular network. Surprisingly, our results show, for the first time, that the construction of vessel-like structures in a Matrigel™ matrix by OECs occurs only during the first hours of the assay, regardless of the presence or not of MMP-9 or a stimulus such as ischaemia, which could not accelerate or slow down the establishment of the network. After this construction phase, the cells were seen in a continuous and dynamic movement in the remodelling phase maintaining the network extension in all experimental conditions. We hypothesize that this observation may respond to the incapacity for stabilization and maturation of newly formed vessels because of the absence of supporting cells as pericytes as described 37. Our results support additional studies to evaluate if the stability of endothelial cells in more mature vascular structures could change the pattern of vascular network remodelling.

In summary, the present study demonstrates that focal cerebral ischaemia triggers the angiogenic responses of EPCs in a mouse model of stroke. Our findings indicate that MMP-9 deficiency impairs the mobilization and angiogenic abilities of EPCs, demonstrating that this protease is necessary for *de novo* vascular network formation in the context of ischaemia. We conclude that MMP-9 modulation may be important for EPC-based therapies to potentiate angiogenesis in the context of ischaemia. Further *in vivo* studies are needed to determine the role of the MMP-9 in angiogenesis in a multiple cell environment and how this metalloproteinase could be modulated, for example, generating cells that overexpress this key modulator of EPCs function.

## Supporting information

Figure S1. Method for culturing EPCs: Mononuclear cells (MNC) were isolated from spleen (mouse) or blood (human) by Ficoll gradient density and seeded in fibronectin (FN)-coated plates with EGM-medium. **Figure S2.** Genotype characterization of wild-type (WT) and knockout (MMP-9/KO) mice. **Figure S3.** Immunocytochemistry of cobblestone-type mouse OECs. **Figure S4.** Representative microscopy images of time-lapse imaging assay of mouse OECs on MatrigelTM matrix at different time-points. **Figure S5.** Gelatin zymography of conditioned media (CM) detected gelatinolytic activity of pro-MMP-9 in media from WT sham and ischaemic EPC. **Figure S6.** Cell viability determined by MTT assay after MMP inhibitors or vehicle treatment. **Figure S7.** Brain vasculature in WT and MMP-9/KO mice after cerebral ischaemia.

Video S1 Twenty-four hours of time-lapse imaging MatrigelTM assay of mouse WT control OECs (1009).

Video S2 Twenty-four hours of time-lapse imaging MatrigelTM assay of mouse WT ischaemic OECs (1009).

Video S3 Twenty-four hours of time-lapse imaging MatrigelTM assay of mouse MMP-9/KO control OECs (1009).

Video S4 Twenty-four hours of time-lapse imaging MatrigelTM assay of mouse MMP-9/KO ischaemic OECs (1009).
