# Supplementary material for: Cerebral ischaemia and matrix metalloproteinase-9 modulate the angiogenic function of early and late outgrowth endothelial progenitor cells
Source: J Cell Mol Med. 2013 Aug 15;17(12):1543–53. doi: 10.1111/jcmm.12116 (PMC3914647; doi:10.1111/jcmm.12116)
Supplement: Supplementary file 1 — Figure S1. Method for culturing EPCs: Mononuclear cells (MNC) were isolated from spleen (mouse) or blood (human) by Ficoll gradient density and seeded in fibronectin (FN)-coated plates with EGM-medium. Figure S2. Genotype characterization of wild-type (WT) and knockout (MMP-9/KO) mice. Figure S3. Immunocytochemistry of cobblestone-type mouse OECs. Figure S4. Representative microscopy images of time-lapse imaging assay of mouse OECs on MatrigelTM matrix at different time-points. Figure S5. Gelatin zymography of conditioned media (CM) detected gelatinolytic activity of pro-MMP-9 in media from WT sham and ischaemic EPC. Figure S6. Cell viability determined by MTT assay after MMP inhibitors or vehicle treatment. Figure S7. Brain vasculature in WT and MMP-9/KO mice after cerebral ischaemia. [file jcmm0017-1543-sd1.docx]

**SUPPORTING INFORMATION**

**SUPPORTING METHODS**

**Genotyping analysis**

To confirm that MMP9/KO mice were homozygous null for MMP9, genomic DNA was isolated from tail samples of selected mice (n=9 MMP9/KO and n=7 WT), and the disruption of the MMP9 gene was confirmed via polymerase chain reaction (PCR). For wild-type mice we used a sense oligonucleotide primer (5’- GTGGGACCATCATAACATCACA -3’) and an antisense oligonucleotide primer (5’- CTCGCGGCAAGTCTTCAGAGTA -3’). For the MMP9/KO mice a neomycin cassette was used as a sense oligonucleotide primer (5´- CTGAATGAACTGCAGGACGA -3´) and an antisense primer (5´- ATACTTTCTCGGCAGGAGCA -3´). Analysis of PCR-amplified products was performed in 1.4% agarose gel in TBE buffer followed by ethidium bromide staining of DNA bands. Five µl of the PCR-reaction product plus 1µl of loading buffer were loaded in the agarose gel and the electrophoresis was run for 20 min at 120 V.

**Permanent focal cerebral ischemia**

All animals were anesthetized with isofluorane via facemask (4% for induction, 2% for maintenance in air, 79%N_2_:21%O_2_; Abbot Laboratories, Spain) and body temperature was maintained at 36.5–37°C using a self-regulating heating blanket and a rectal probe. Mice eyes were protected from corneal damages during surgery using an ophthalmic lubricating ointment (Lipolac^TM^, Angelini Farmaceutica, Spain). An incision was made between the left eye and ear under an operating microscope (Leica MS5, Switzerland) and the temporal muscle was cut and divided exposing the left lateral aspect of the skull. The MCA was identified through the semi-translucent skull and a small burr hole (1-2 mm diameter) was made using a high-speed microdrill at the level of the inferior cerebral vein to expose the M1 portion, leaving the dura intact. Saline was applied to the area throughout the procedure to prevent heat injury. Cerebral blood flow (CBF) was measured continuously by laser-Doppler flowmetry using a flexible fiberoptic (0.5 mm diameter; Moor Instruments, UK) placed directly on the top of the parietal branch of the M1 bifurcation beginning 5 min before MCAO, during and after the electro-cauterization. Using a micromanipulator holding a 30G needle (0.4mm diameter) the MCA was compressed and a decrease in the CBF was ensured. Therefore, using a small vessel cauterizer (Change-A-Tip^TM^, Aaron Medical, USA) the MCA was permanently occluded by indirect electrocoagulation thorough the 30G needle. Then, the muscle was replaced and the skin was sutured using 5-0 silk suture and magnesic metamizol (400mg/kg) was administered subcutaneously right after the procedure as analgesic. The duration of anesthesia in all animals was less than 30 min. Only mice that showed decreased CBF below 75% from baseline were used for further experiments.

Sham animals underwent all surgical procedures with the exception of the MCA occlusion (MCAO). Six, 24 or 72 hours after the ischemia, or after 24 hours of the surgery in sham group, mice were sacrificed.

**Mouse Spleen Endothelial Progenitor Cell Cultures**

Mouse early EPCs enriched population was obtained as described. Spleens from WT and MMP9/KO mice were obtained at 6, 24 or 72 hours after ischemia and in sham animals after 24h. A pool of 2 spleens was used for each cell culture. Spleens were mechanically minced, placed at 37ºC for 15 minutes in a 1mM EDTA solution and run thorough a 40-mm nylon membrane to obtain a cell suspension. Mononuclear cells (MNCs) were obtained by density gradient centrifugation with Ficoll-Paque Plus (GE Healthcare, Sweden), shortly washed with red blood cells lysis solution (150 mmol/L NH_4_Cl, 10 mmol/L NaHCO_3_ and 0.1 mmol/L EDTA in distilled water) and gently washed with complete endothelial growth medium-2 (EGM-2; Clonetics®, CA, USA), which is composed of endothelial cell basal medium (EBM) containing 20% fetal bovine serum (FBS), human epidermal growth factor (hEGF), vascular endothelial growth factor (VEGF), human basic fibroblast growth factor (hFGF-B), insulin like growth factor 1 (R3-IGF-1), GA-1000 (gentamicin and amphoterecin-B), heparin, hydrocortisone and ascorbic acid. Isolated MNCs were finally resuspended in EGM-2 and 10^7^ MNCs were seeded on fibronectin-coated 12-well cell culture plates (4 replicates per sample) and incubated in 5% CO_2_ at 37ºC. Under daily observation, first media change was performed 2 days after plating and, thereafter, media was changed every 2/3 days. Expanding cell-colonies appeared between days 10 to 20. This method has been previously described in other studies to study EPCs counts and allowed us to maintain the cells in culture to further obtain outgrowth endothelial cells whereas other techniques such as flow cytometry do not allow maintaining the EPCs in culture [1-3].

A total of 52 cell cultures were performed from 104 mice (2 spleens each) and images from five representative fields were taken at 200x on day 5.

**Human Blood Endothelial Progenitor Cells Cultures**

Twenty ml of blood were diluted (1:1) with PBS containing 2% FBS. Afterwards MNCs were obtained by Ficoll gradient as described for mouse EPCs, cells seeded in fibronectin-coated plates and grown to obtain OECs. All procedures were approved by the ethics committee of our institution and were conducted in accordance with the Declaration of Helsinki.

**Immunocytochemistry**

Standard EPC phenotyping was performed in palisade-type OECs from WT and MMP9/KO mice and cobblestone-type human OECs for von Willebrand factor, KDR and CD133 antigens. Cells were fixed with cold 4% paraformaldehyde for 20 min at room temperature (RT) and washed with PBS. Afterwards, cells were permeabilized with 0.3% triton X-100 for 5 minutes, blocking buffer (1% BSA and 5% goat serum) applied for 1 hour and incubated with primary antibodies rabbit anti-von Willebrand factor (1:100, Sigma-Aldrich, USA), mouse anti-KDR/VEGFreceptor 2 (1:100, Sigma-Aldrich, USA) and rabbit anti-CD133 (1:50, Santa Cruz, USA) overnight at 4ºC. Goat anti-rabbit Alexa Fluor 488 or Alexa Fluor 568 (Invitrogen, USA) were used as secondary antibodies at RT for 1 hour. Samples were finally mounted in Vectashield^TM^ with DAPI (Vector Labs, USA) to counterstain cell nuclei. Negative controls received identical treatment except for the primary antibody.

***In vitro* vessel formation**

All experiments were performed with OECs between passages 4 to 8 for mouse cells and between 7 and 12 for human cells. Briefly, 24 well-plates were coated with 200 µl cold Matrigel^TM^ growth factor-reduced basement membrane matrix and allowed to solidify at 37ºC for 30 min. Afterwards, 4x10^4^ cells per well for mouse and at 6x10^4^ cells per well for human cells seeded into Matrigel^TM^-coated wells in basal media (without factors and FBS) or basal media plus treatment as detailed above. For MMP9/KO cells treated with CM from WT cells, EBM-2 was replaced for CM (obtained after 24 hours incubation in EBM-2) from the same experiment. Cells were incubated at 37ºC during 24 hours. Each assay was performed in duplicate and the number of complete rings (circular vessel-like structures) and the total tube length (perimeter of the complete rings) were counted by ImageJ software (NIH, MD, USA) by an investigator blinded to the treatment in 6 representative fields (100x) per well. Mean values were used for comparisons between cell types while experimental treatments with MMP inhibitors, CM or recombinant MMP9 were expressed as percentage of the non-treated group.

**Cell Viability**

Cell viability assay was performed to asses the toxicity of the treatments applied to to the OECs. Measurement of the reduction of 3-(4,5-dimethylthiazol- 2-yl)2,5-diphenyl-tetrazolium bromide (MTT) to produce a dark blue formazan product was performed to assess the integrity of mitochondrial function as a measure of cell viability. Briefly, 4x10^4^ OECs were seeded on fibronectin-coated 24-well plates in EGM-2 medium. Twenty-four hours later the cells were washed with PBS and treated with EBM (basal medium) plus pharmacological treatment to inhibit MMPs: the broad spectrum GM6001 and the specific MMP9 inhibitor I or their corresponding vehicles (in mouse cells: 2 mM Tris, 6 mM NaCl, 0.8 mM CaCl_2_ for GM6001 and 14.6 mM DMSO for MMP9 inhibitor I; and in human cells: 4 mM Tris, 12 mM NaCl, 1.6 mM CaCl_2_ for GM6001 and 146 mM DMSO for MMP9 inhibitor I) added to the same final concentration of the Matrigel^TM^ assay. The reduction of MTT was measured after 24 hours of treatment. Absorbance was measured at 590 nm and each sample was measured per duplicate to obtain a mean value and results for treatments are expressed as a percentage of the control non-treated group.

**Gelatin Zymography**

Conditioned mediums of WT control and ischemic EPC cultures were collected and 0.5 ml were concentrated with 10K membrane centrifugal filters (Amicon Ultra-0.5ml 10K Ultracel, Millipore, Germany), obtaining from 30 to 50 µl of concentrated media. Twenty microlitres of concentrated conditioned media were loaded and separated by 10% tris-glycine gel with 0.1% gelatin as substrate, washed with renaturing buffer (Invitrogen, USA) for 90 minutes (2x45 min) and further incubated with developing buffer (Invitrogen, USA) at 37ºC for 48 hours. Finally, the gels were stained with 0.5% Comassie blue R-250 for 1 hour and then appropriately distained (3 x 20 minutes). Molecular weight markers and human MMP2 and MMP9 (Chemicon, MA, USA) recombinant proteins were used as standards.

**Brain vasculature quantification**

A new group of animals (8 WT and 8 MMP9/KO mice) were subjected to permanent focal cerebral ischemia (4 animals of each genotype) or sham surgery. After 21 days, mice were injected intravenously with 80 µg of Dylight 594-labeled tomato lectin (*Lycopersicon esculentum*; Vector Laboratories, CA, USA) and sacrificed by cardiac perfusion of 4% paraformaldehyde under deep anesthesia after 10 minutes. The brains were removed, post-fixed overnight in 4% PFA and cryoprotected with 30% sucrose in PBS for 24 hours. Afterwards, the brains were frozen and embedded in OCT before storage at -80ºC. Twelve-μm thick coronal sections were collected from anterior (+1 to +0.2 bregma) and posterior (-1.7 to -2.3 bregma) areas including the lateral ventricles and the hippocampus respectively. Sections were thaw at room temperature for 30 minutes, transferred to PBS for hydratation and slices were mounted in Vectashield^TM^ with DAPI to counterstain cell nuclei. Four images (100x) of the peri-infarct cortex of ischemic mice or the respective area in sham animals were taken, and the total area of lectin positive vessels was calculated with standard computer-assisted image analysis technique (Image J free software, NIH) by an investigator blinded to the treatment. Results are expressed as the mean vessel density of the 4 images in each area.

**SUPPORTING REFERENCES**

**[1] Rosell A, Arai K, Lok J*, et al*. Interleukin-1beta augments angiogenic responses of murine endothelial progenitor cells in vitro *J Cereb Blood Flow Metab.* 2009; 29: 933-43.**

**[2] Hayakawa K, Pham LD, Katusic ZS*, et al*. Astrocytic high-mobility group box 1 promotes endothelial progenitor cell-mediated neurovascular remodeling during stroke recovery. *Proc Natl Acad Sci U S A.* 2012; 109: 7505-10.**

**[3] Werner L, Deutsch V, Barshack I*, et al*. Transfer of endothelial progenitor cells improves myocardial performance in rats with dilated cardiomyopathy induced following experimental myocarditis. *J Mol Cell Cardiol.* 2005; 39: 691-7.**

**SUPPORTING FIGURES AND FIGURE LEGENDS**

**Supporting Figure 1.**

**
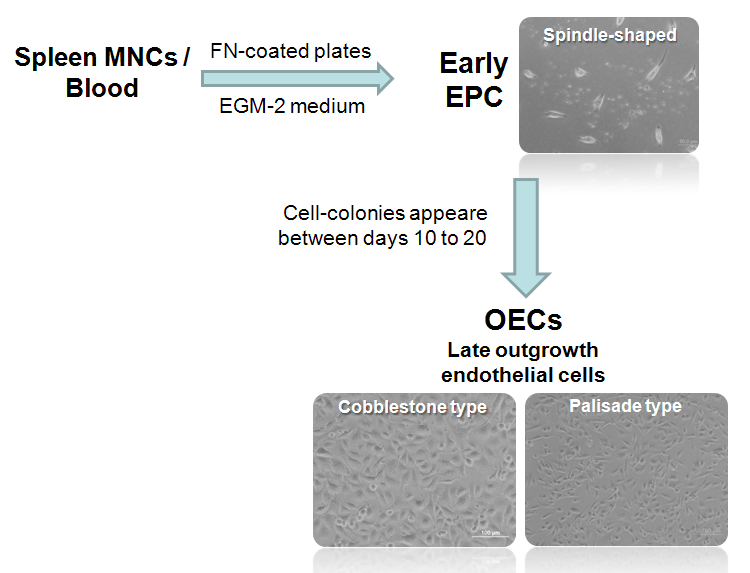
**

**Supporting Figure 1.** Method for culturing EPCs: Mononuclear cells (MNC) were isolated from spleen (mouse) or blood (human) by Ficoll gradient density and seeded in fibronectin (FN)-coated plates with EGM-2 medium. Early EPCs (also called circulating angiogenic cells, CAC) were counted as “spindle-shaped” cells. Colonies of OECs appeared as either cobblestone or palisading type.

**Supporting Figure 2.**

**
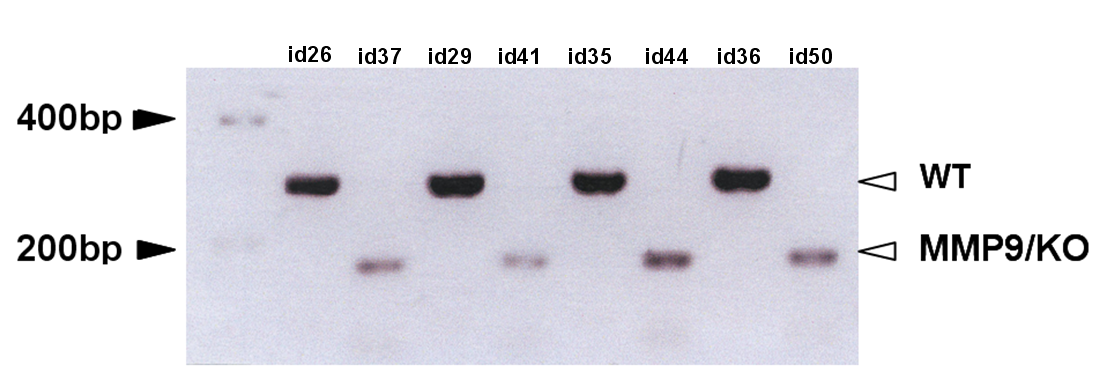
**

**Supporting Figure 2: Genotype characterization of wild-type (WT) and knockout (MMP9/KO) mice.** Agarose gel electrophoresis showing PCR products after DNA amplification of part of the MMP9 gene in WT mice (277bp band) and the disrupted MMP9 gene by neomycine resistance in KO mice (172bp band).

**Supporting Figure 3.**

**
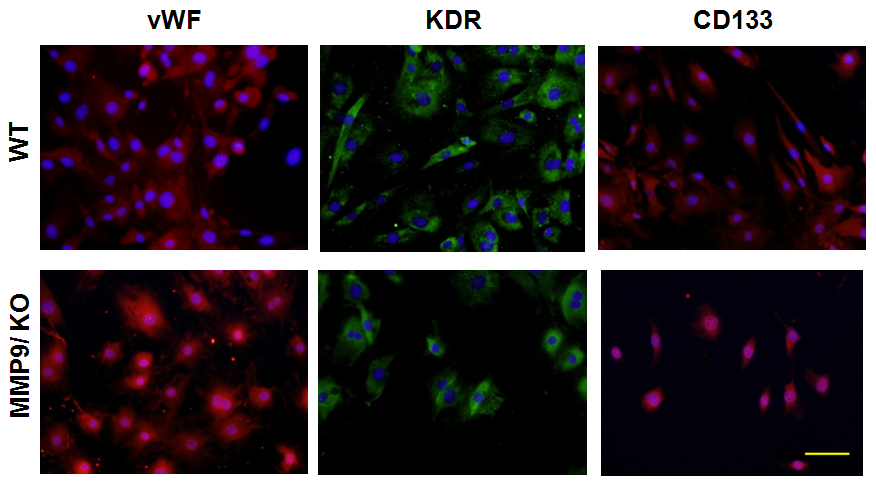
**

**Supporting Figure 3. Immunocitochemistry of cobblestone-type mouse OECs.** Immunofluorescent staining of OECs showing positive signal (red or green) for von Willebrand factor (vWF), KDR and CD133 (bar=50µm) in cells obtained from WT or MMP9/KO mouse.

**Supporting Figure 4.**


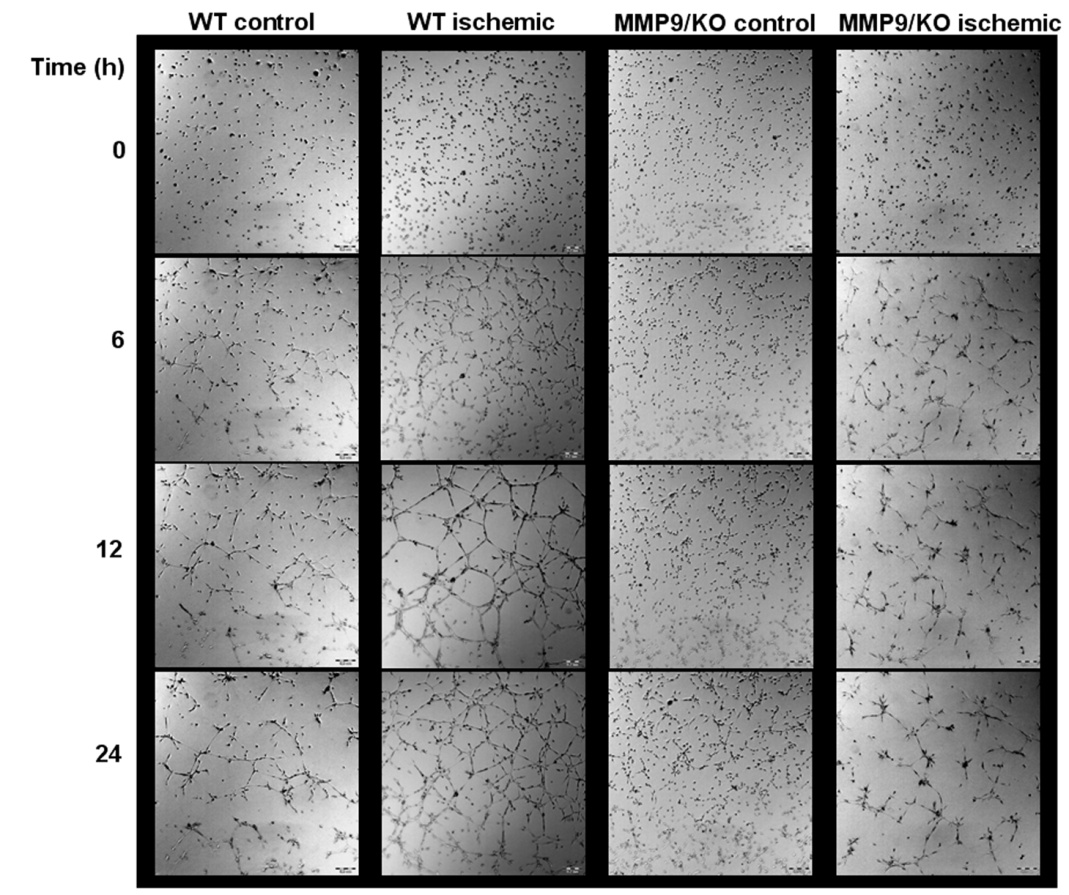


**Supporting Figure 4. Representative microscopy images of time-lapse imaging assay of mouse OECs on Matrigel™ matrix at different time points**. (bar=200µm).

**Supporting Figure 5.**


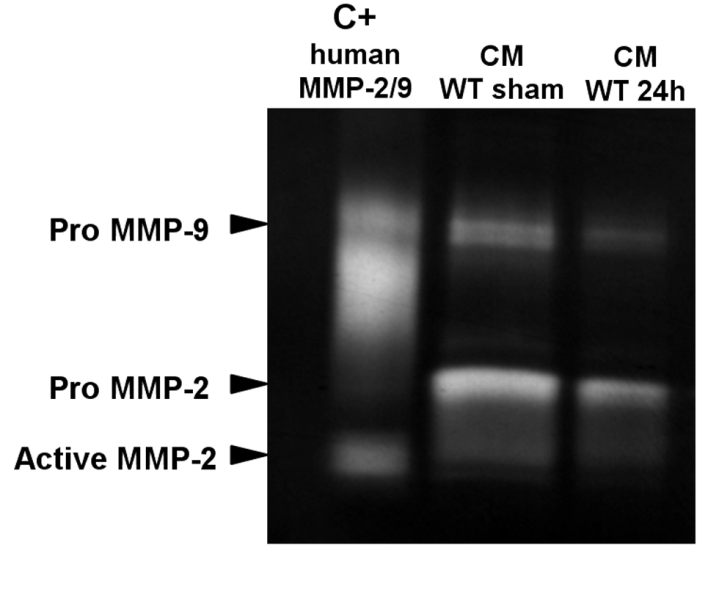


**Supporting Figure 5:** Gelatin zymography of conditioned media (CM) detected gelatinolytic activity of pro-MMP9 in media from WT sham and ischemic EPC.

**Supporting Figure 6.**

**
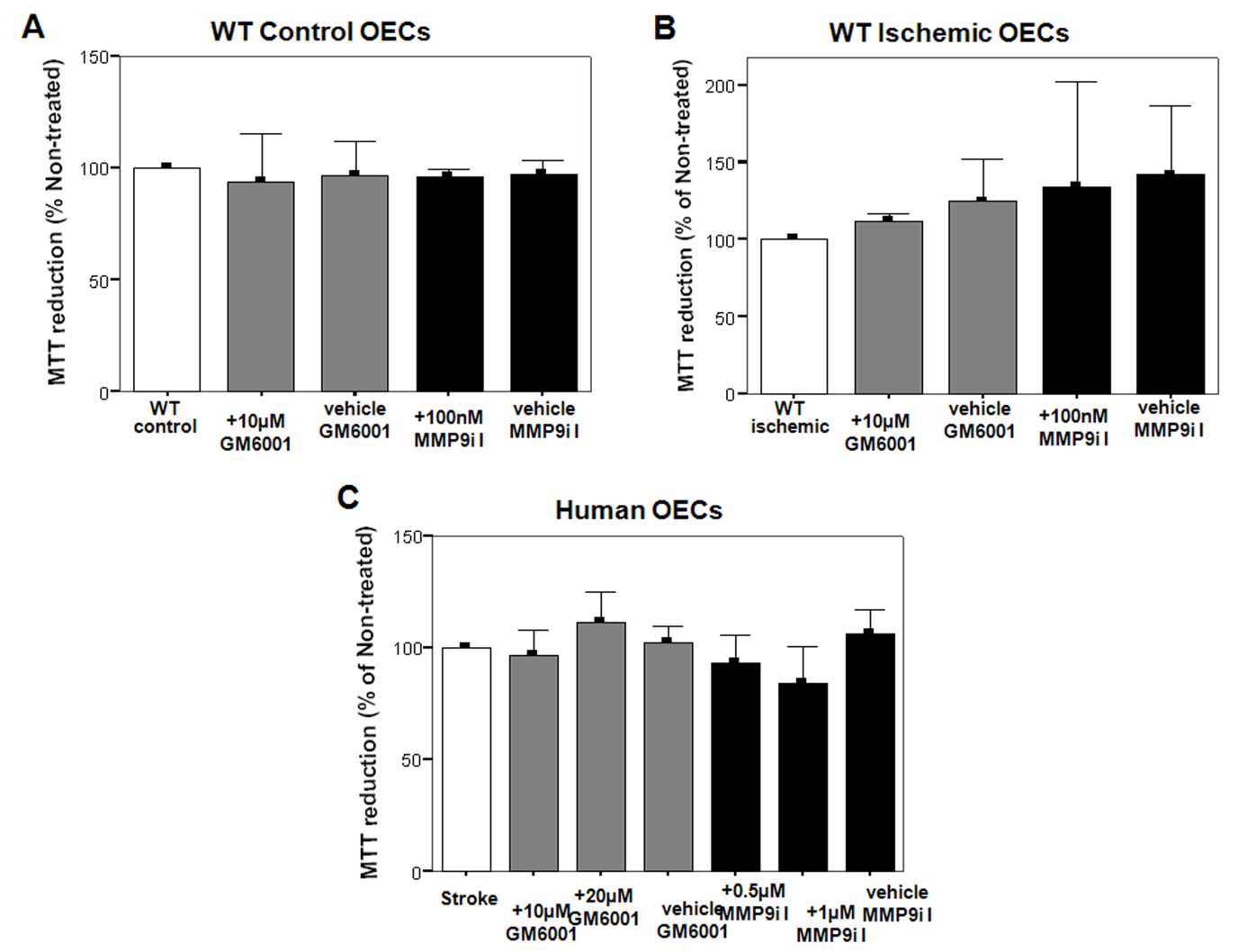
**

**Supporting Figure 6. Cell viability determined by MTT assay after MMPs inhibitors or vehicle treatment.** Bar graphs represent cell viability for OECs from WT control mouse **(A)**, WT ischemic mouse **(B)** and human control subjects **(C)**; n=4 independent experiments per group. Results are expressed as mean±SD and referred to the non-treated group. No significant differences were found.

**Supporting Figure 7.**


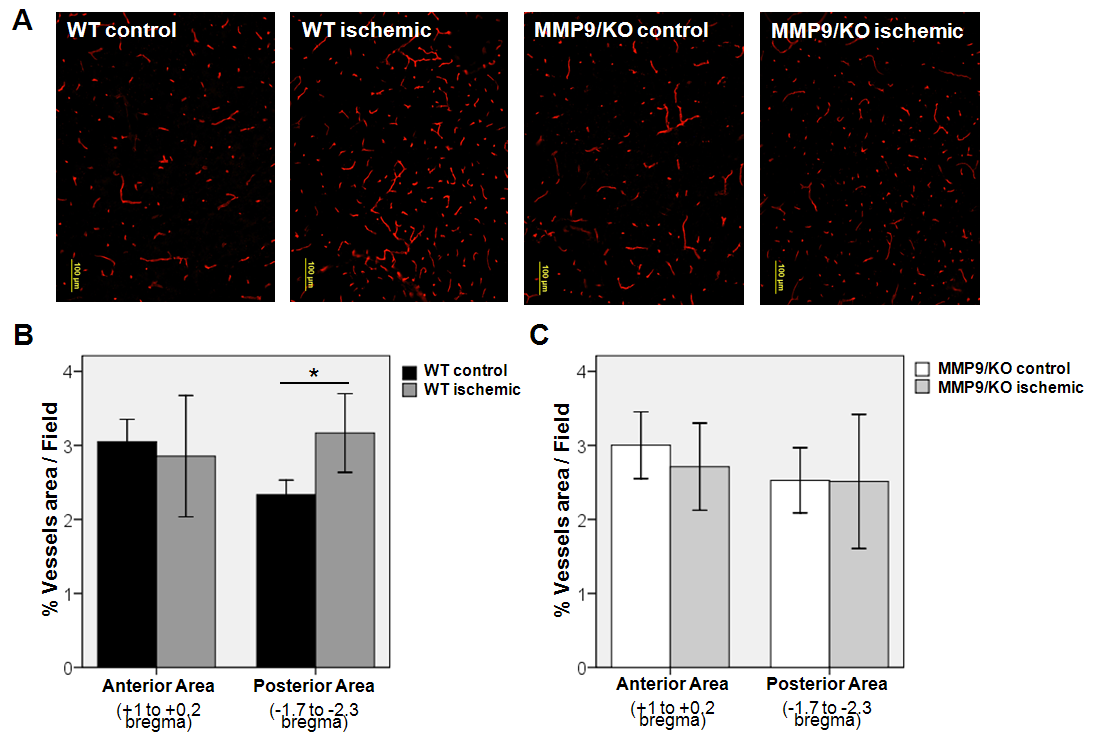


**Supporting Figure 7. Brain vasculature in WT and MMP9/KO mice after cerebral ischemia.** Functional blood vessels stained after lectin perfusion were quantified in the ipsilateral peri-infarct cortex 21 days after ischemia and in corresponding areas of sham mice. **A)** Representative micrographs (10X objective) of lectin staining (bar=100µm). Bar graphs show that only WT mice increased vessel density compared to non-ischemic animals in the posterior areas **(B)** while no differences were observed in MMP9/KO mice **(C)**. Data represents mean±SD, *p<0.05 (n=4 mice per group).

**LEGENDS FOR VIDEO FILES**

**Supporting Video 1**: Twenty-four hours of time-lapse imaging Matrigel^TM^ assay of mouse WT control OECs (100x).

**Supporting Video 2**: Twenty-four hours of time-lapse imaging Matrigel^TM^ assay of mouse WT ischemic OECs (100x).

**Supporting Video 3**: Twenty-four hours of time-lapse imaging Matrigel^TM^ assay of mouse MMP9/KO control OECs (100x).

**Supporting Video 4**: Twenty-four hours of time-lapse imaging Matrigel^TM^ assay of mouse MMP9/KO ischemic OECs (100x).
